# Insights from expert coaches on technical performance evaluation in rowing: a pilot study

**DOI:** 10.3389/fspor.2024.1448797

**Published:** 2024-10-10

**Authors:** Erik Baumann, Michael J. Schmid

**Affiliations:** Institute of Sport Science, University of Bern, Bern, Switzerland

**Keywords:** coaching, coaches’ eye, expertise, rower, technical skills assessment

## Abstract

**Introduction:**

Since rowing became an Olympic sport in 1900, rowers have made significant performance gains, partly attributed to increased research, training, and competition knowledge. Rowing technique and biomechanics play an essential role in rowing performance. While some aspects can be quantified with modern tools, coaches' expertise remains essential for technical performance evaluation. Coaches often play a pivotal role in identifying and correcting technical faws; however, novice and intermediate coaches may struggle. This study examines how expert-level rowing coaches assess the technical performance of athletes during on-water rowing.

**Methods:**

Four current and former national team coaches were interviewed using semi-structured interviews. The repertory grid technique was employed to explore their initial foci when assessing the rowing technique. The acquired data were content-analysed and listed in a summarising table.

**Results:**

We have detailed both the positive and negative aspects of rowing technique identified by these coaches. Three overarching themes were identified: *perceived force application, perceived movement precision, and perceived rhythm and timing*.

**Discussion:**

Examining the categories revealed that the coaches with a shared federation background exhibited a relatively high level of similarity in their initial foci. However, looking into their subjective aspects revealed considerable differences. This leads to the hypothesis that a broad spectrum of unique coaches' criteria can lead to the same or similar technique executions from their athletes.

## 1 Introduction

Rowing has been an Olympic sport since the second modern Olympic Games in Paris in 1900. At the 2024 Olympic Games, athletes participated in fourteen medal events—seven each for female and male athletes. Rowing can be divided into two main disciplines: sweeping and sculling. In sweeping, the crew is divided equally into starboard and portside rowers. Every crew member holds on to an oar with both hands. The arm closer to the pin/oarlock (where the oars are attached to the boat's riggers) is called the “inside arm,” and the one further away is the “outside arm” (1). In sculling, however, every rower holds on to two sculls, which are shorter and lighter than the oars. The standardised 2,000-m course was introduced in the 1912 Games in Stockholm. Since then, rowing performances have improved drastically, with modern rowers racing the 2,000-m races significantly faster than in the early days of the modern Olympics.

According to Nolte (2), these incredible increases in sports performance are achieved in three ways. First, today's athletes generate more energy (i.e., they are fitter); second, they can use their energy more efficiently; and the third way is the combination of the previously mentioned strategies. Modern exercise science and physiology knowledge helps coaches and rowers achieve greater overall fitness. However, in this paper, we will mainly explore the efficiency of the rowing stroke by looking at technique, a topic discussed in the field of rowing biomechanics.

In rowing, the motor task posed to the athletes may be described as closed, as Gentile (3) defined, since the environment is mostly standardised to a 2,000 m flat water course. Nonetheless, rowing performances are influenced by environmental factors like wind or waves. Like in most endurance sports, the technique in rowing is a cyclic movement and, therefore, is repeatedly performed throughout every race and training. Consequently, coordinative abilities do not have to be as diverse as those required by athletes in sports such as football or gymnastics. Nevertheless, technique constitutes a critical component required for boat speed, especially when facing opponents with similar physical and mental abilities. Technical proficiency can give crews a clear advantage over less skilled competition (4). While physical fitness is often the central consideration for coaches when it comes to selection/evaluation, the technique might be an underestimated factor for some teams.

Indoor rowing machines like the Concept2 Indoor Rower (Morrisville, Vermont, USA) are used as a popular tool to evaluate the physical fitness of athletes (5). Mikulić et al. (6) assessed the correlation between 2,000 m ergometer results and final rankings at the 2007 World Rowing Championships in Munich, Germany. They determined that ergometer scores were not a sufficient predictor for the final rankings. These results suggest that sculling and sweeping have an increased demand for technique compared to rowing machines such as the Concept2 Indoor Rower. Unlike ergometer rowing, on-water rowing requires balance, movement economy, and boat speed maintenance during the recovery (6). It is worth mentioning that it has been suggested that ergometer scores can be improved by applying proper technique (7, 8). Therefore, technique seems to play an important role in ergometer rowing likely has a more significant impact in on-water rowing.

The frequent use of on-water technique training sessions in high-level rowing programs supports the importance of technique in the sport (9). Good technique can lead to efficient power transmission (10), which can lead to increased boat speed. Consequently, rowers without proper technique waste power and are less effective. Additionally, an improper execution of the technique leads to more injuries than a proper technique (10). The proper technique can be learned through technical training (4). For this, the rowing stroke is divided into smaller, fundamental components. To tackle technical issues or to master certain aspects of rowing, various drills can be used and reintroduced to the full stroke.

Following Hossner et al. (11), cyclic sports movements, such as rowing, can be divided into a main sub-action and an auxiliary. Of the four parts of the rowing stroke (i.e., drive, recovery, catch, and finish) (12), the main sub-action is the “drive.” It starts when the blades are placed in the water at the “catch position,” where the handles are closest to the stern of the boat (2). This position is reached when the knees reach their minimum angle, the upper body is leaned forward, and the arms are extended. As soon as the oar blade is anchored in the water, the rowers push themselves and their boat past the blades. The rowers use their blades and the resistance of the water to create an acceleration of the shell. The drive ends at the “finish” position (also called the “release” position), where the handles come closest to the bow of the boat (2). Accordingly, the rower's legs are extended, the upper body is leaned backwards, the arms are bent, and the handles are closest to the rower's body. At this point, the blades are extracted from the water and then feathered (turned 90°, parallel with the water). After that, the auxiliary sub-division, “the recovery,” begins. The drive's movements are repeated in reversed order and direction: the arms are extended, the upper body is angled forward, and the knees go back to their minimum angle. The rowers square their blades (turning them back 90°) during the recovery so that they are ready for the entry when the rower reaches the catch position at the end of this movement.

Sport-specific technical skills are considered a vital performance resource. Therefore, the coach's ability to identify flaws is an important skill, a phenomenon called “coaches’ eye” or “coach's eye” (13). This ability manifests itself differently in novice and expert coaches. For example, Schempp and Woorons (14) found that while expert and novice coaches picked up on a similar number of cues during video assessment and slide recall of a tennis practice match, the relevance of the cues noticed by the two groups varied significantly. While expert coaches focus on cues of high importance for technique performance, novice coaches mention less critical features such as the player's apparel or background features. Expert coaches stand out and achieve success as a result of their vast and profound domain-specific knowledge in the field (15). Their feedback increases the rowers’ potential to optimise technical performance. The relevance of coaches’ feedback method is of tremendous importance because it gives crews and individual athletes immediate information about their performance (16). However, the interpretation of technical errors varies, as the rowing stroke is approached from many subjective points of view. Information gathered from biomechanical feedback tools can be helpful for objective analysis of the efficiency of the displayed (16), but these methods might be too expensive or difficult to analyse for certain crews, and, therefore, they cannot replace the coaches’ eye. An additional challenge for coaches is that they might experience difficulties putting into words what exactly they are looking for in athletes (17).

Coaching is a decision-making process (15), so coaches must deeply understand athletes and their potential to select crews and coach them to succeed. Besides anthropometric, physiological (18), and psychological factors (19), rowing technique (20) is typically used to assess a rower's performance potential. However, the ideal rowing technique is influenced by individual factors like athlete anthropometrics and physiology (21). A shorter and lighter person might have to row differently from a taller and heavier athlete to reach their potential. In research, there are no data on ideal biomechanical strokes for any particular rower with their anthropometric characteristics (21). What works best for one rower might not be the ideal technique for a rower with a different physique.

Technical evaluations are often done by eye or video analysis (22). However, they often lack objectivity since there is no established definite link between technique and boat speed (23). A clear guideline for developing coaches is presumably missing because the knowledge of “perfect technique” is limited.

Furthermore, Lath et al. (13) found four main characteristics of the coaches’ eye: intuitive, subjective, holistic, and experience-based. Intuitive refers to the reliance on gut feeling and implicit knowledge in decision-making; subjective highlights the influence of individual preferences and experiences; holistic emphasises the comprehensive assessment of an athlete; and experience-based underscores the importance of accumulated coaching experience in recognising patterns (13). Especially, a lack of experience in novice and developing coaches might lead to problematic coaching approaches and choices. The literature suggests various ways in which the skills of inexperienced coaches can be improved. Soper and Hume (21) mention that novice and developing coaches can be guided by experts in their understanding of skilled movement. For example, a coach developer can promote the exploration and learning of coaches by “guiding a coach's attention to critical features and information in the environment” [(24), p. 614]. According to Nolte (25), a qualitative approach to the rowing technique can develop the perception of a model rowing technique (e.g., what is crucial), helping a coach notice observed deviations in the performed technique.

Therefore, this study aimed to explore how expert coaches assess on-water rowing technique to give novice and intermediate coaches who seek to develop their “coaches’ eye” some cues, ideas, and examples of what to look for in athletes to ensure effective rowing technique and thereby improving their assessment and coaching.

## 2 Materials and methods

Given the limited knowledge of the subjective technical criteria of expert coaches, a qualitative methodology with an inductive approach was deemed appropriate for this study. We aimed to assess these criteria as nuanced and linguistically accurate as possible. Following Kelly (26), we assume that every expert has an individual system of constructs that influences their perception and judgment. Thus, the constructs used by rowing coaches form their subjective technique criteria, which can be regarded as their personal technique model for rowers. In order to understand these constructs, the present study adopts a critical realist perspective, which assumes both ontological realism and epistemological constructivism (27). We assume that while there is a reality independent of our attempts to recognise it, our knowledge of this reality is fallible, theory-laden, and can only be acquired through our discourses (28). Moreover, our research aims to address a practical challenge for novice and intermediate level coaches, ensuring relevance and applicability to everyday coaching practice [see also (29)].

### 2.1 Participants

The inclusion criterion for participation was coaching experience at the national rowing team level for at least 5 years. All coaches have worked in a leading position at the Swiss Rowing Federation at some point in their careers. Four expert-level coaches (one female, three male) with 20–35 years of coaching experience in rowing (*M* = 26.3 years, *SD* = 5.4 years) were recruited and interviewed for this study. They have completed the highest coaching education programme. Moreover, they have also competed in rowing competitions at the international level. All four coaches have worked with several world championship and Olympic medallists. At the time of the interview, the coaches were either still coaching internationally or had moved on to other jobs within the rowing community.

### 2.2 Procedures

Before the data collection began, the institutional research ethics committee of the University of Bern approved the study and its methodological approach. At the beginning of the expert interview, the interviewer clarified the purpose of the study. After the informed consent form was signed, the interview started. The interviews were held online on a video call. Three interviews were held in German, and one was in English.

Following Jokuschies et al. (30), who analysed the subjective talent criteria of football coaches, the present study was also based on the repertory grid technique (31). This involves characterising a construct using two poles (i.e., construct and contrasting pole). Faber et al. (32) used a similar approach to develop a tool to assess technical skills in table tennis players. Therefore, the semi-structured interview (see Supplementary Material) guide of Faber et al. (32) was adapted for the rowing technique. A pilot study was conducted with a club-level coach with experience at the national team level to evaluate the quality of the interview and for the interviewer to test the interview. The interview guide delivered the required data in this trial run. Some minor adjustments to the introduction were made to avoid ambiguity.

Initially, the first task was to find out about the coaches’ experience in rowing as an athlete and as a coach. This is helpful in determining their level of experience and expertise. In the second part of the interview, the questions aimed to provoke insightful perspectives on which aspects of the rowing technique these coaches consider particularly relevant. This was achieved by asking the coaches about their initial focus when looking at a crew or single sculler for the first time (i.e., “Imagine yourself watching a newly formed crew of highly skilled rowers [in the context of a club or national team] or a single sculler that you have not seen rowing before. Your goal for this session is to evaluate their technical skill level. What are some things you would consider?”).

After accumulating the most relevant aspects, the participants were asked to describe perfect execution as well as flawed execution of these aspects (i.e., “If you consider …; what is the perfect performance? And what would be the worst or flawed performance? Explain, clarify, describe an ideal execution of this aspect”).

For the data extraction, the interviews were transcribed in their original language. The coaches’ cited passages are translated into English if required. All four interview recordings took between 40 and 46 min.

### 2.3 Data analysis

The answers given in the interviews were evaluated using an inductive content analysis according to Patton (33). In the initial step, the interviews were transcribed. Secondly, the criteria and the positive and negative executions of the technique given by the coaches were extracted and listed. Each set of positive and negative criteria was assigned one overarching name: we refer to these as *aspects*. The extracted aspects were then analysed for similarities and differences. According to this expert analysis of the primary author, who has an extensive background in rowing, each aspect was assigned to one of the extracted *categories*. The second author, who is also extensively experienced with rowing, checked whether the categories were fitting and if the criteria were assigned to the adequate category. The overall results were also presented to the interviewees, which gave them a final chance to check the data and the author's interpretation of the interview data. During this second round of discussion, the interview data were finalised with the help of the expert coaches.

We considered the three types of validity provided by Maxwell (34) to ensure validity. Descriptive validity is secured thanks to accurate transcriptions and a fitting translation of the quotes by a fluent transcriber in German and English. We sent our results to the interviewees to promote interpretive validity. Finally, to increase theoretical validity, in various dialogues, the authors discussed claims and discussed whether the explanation are suitable.

## 3 Results

In our analysis, we have identified and developed three overarching categories of technical aspects (see Table 1): (a) *perceived movement precision*, (b) *perceived force application*, and (c) *perceived rhythm and timing*. The spatial category *perceived movement precision* raises topics concerning the body's or the blade's position during the stroke. The category *perceived force application* is concerned with how (and if) the rowers use their power to create boat propulsion. The last category, *perceived rhythm and timing,* concerns the temporal execution of the movement. While the positions might be adequate, a good technique relies on a well-timed execution of various parts of the stroke.

**Table 1:** Subjective technique criteria and assigned category per coach in order of occurrence during interviews.

	Technique criteria	Poles		Assigned category
		Positive	Negative	
Coach A	Correlation between body movement and boat movement	Hands move towards the body according to boat speed, Hands move away from the body according to boat speed, Fluid movements	Athletes move faster but the boat does not go faster, Car in wrong gear, Hands go out too slow or too fast	Perceived force application
	Catch	Pick up the boat at the right moment (with the feet), No more movement with the upper body and arms after initial preparation, Catch at the furthest point	Segmented/non-continuous movements	Perceived rhythm and timing
	Blade during drive	Moving the boat, No foam around the oar, Oar bend, Hands are led to the rower's bodies instead of down to their laps, Continuous acceleration	Blade moving through the water, White water around the oar, No oar bend, Oar coming out of the water while arms are still moving towards their body, Jerky movement	Perceived force application
	Rhythm	2:1 recovery-to-drive-ratio (during steady-state rowing), Looks like the crew uses the right gear	More time spent on drive than recovery	Perceived rhythm and timing
	Phase structure	Legs, hips, arms (drive) and arms, hips, legs (recovery), Parallel movement of the torso during the leg-drive (keeping upper body and arms in catch position as long as possible)	Upper body opens right at catch, Hip extension and arm pull simultaneous	Perceived movement precision
Coach B	Catch	Immediate oar bend, Back splash, Immediately connected (body, boat, and blade)	Straight oar, Dipping upper body between the legs, The stern is dipping at the catch	Perceived rhythm and timing
	Immediate leg drive after catch	Oar bend, Sequence progression: phases are initiated as late as possible and as early as needed	Opening the upper body right after the catch instead of picking up the boat with the legs	Perceived force application
	Blade during drive	Oar bend, Lower back straight (position like the deadlift), Blade covered until the end of the arm pull	No oar bend, Rounded back, White water around the blade	Perceived force application
	Release for recovery phase	Tapping down the handle and then changing directions, Preparation (hands away, hips, etc.), Very balanced boat after simultaneously tapping down	Changing directions while getting the blade out of the water	Perceived movement precision
	Back shape	Deadlift, Tall, pelvic tilt	Rounded back, Arms bent	Perceived movement precision
Coach C	Synchronicity	Blade work: the blades go in and out of the water at the same time	One blade is in the water while the others are/other is out of the water	Perceived rhythm and timing
	Length	Depends on boat category: faster boats require more work in front of and less work after the oarlock height, Compact at catch: small angles between shins and quads, and quads and torso, Wide open arms at catch	Losing body tension to lean forward, Not using the full potential slide length	Perceived movement precision
	Body tension	Work with the legs at the catch, Slightly hollow back (lower back has the same position as in a front squat)	Round lower back, Beach chair position, Dipping the body at the catch or bum shoving, Sitting in the boat “like a cooked spaghetti”	Perceived force application
	Phase structure	Stacking up the momentum created throughout different parts of the stroke, Keeping the pressure on the blade throughout the stroke (continuous speed)	Significantly overlapping the phases or only starting the next phase when the previous phase is completed	Perceived movement precision
Coach D	Connection at the catch and leg drive	Thinking about putting the blade in while still going forward, Squaring blade early to be ready for its entry, Sweeping: handle height is controlled by the outside hand, Placement and then leg drive	Going to the front and then putting the blade in, Starting the leg drive without having the blade in the water, Sweeping: catching with inside arm	Perceived movement precision
	Timing	Looking at blades: they are going in together, Athletes follow the person in front of them (by looking at their necks)	Blade positions vary, Sculling: right and left side oar are not moving together	Perceived rhythm and timing
	Phase structure	Sequencing depends on the agreed upon technique model		Perceived movement precision

The categories were assigned based on the coaches’ descriptions and not on the extracted title of the technique criteria. This means that while some coaches might have mentioned the same or a similar part of the stroke as a subjective technique criterion, their focus determined the assigned category of an aspect. Therefore, some aspects with similar titles were assigned to different categories. In Table 2, the data are quantified. It is noteworthy that the categories are repeatedly mentioned throughout the four interviews.

**Table 2:** Number of subjective technique criteria From all four coaches by assigned categories.

Coach					
Assigned category of technique criteria	A	B	C	D	Total
Perceived movement precision	1	2	2	2	7
Perceived force application	2	2	1	0	5
Perceived rhythm and timing	2	1	1	1	5
Total	5	5	4	3	17

Although several coaches mentioned related criteria, every coach had a different list of aspects. Between three and five aspects were listed per coach. From these data, the aspects were assigned to one of three categories. In the following sub-sections, a representative aspect of each category is examined. Coaches gave their subjective criteria and later described the perfect and flawed execution of these criteria.

### 3.1 Perceived movement precision

The first category, *perceived movement precision*, includes aspects where a good performance relies on movement precision. Various technical aspects relate to how an athlete moves certain body parts and even the oars during the stroke. In the first quote, Coach D lists one of his technique criteria:

So, what I look at first of all when I look at a crew is: are they actually pushing the legs? So that's probably the most critical thing when the legs are connected to the blade because it's the most powerful part of the stroke and the moment you are not pushing your legs when you're bum-shoving or you use your back too much … That's the first thing I look at.

In the following quote, Coach D describes what he looks for to see if the rower executed the aspect correctly:

Yeah, the number one thing is how they place the blade in. Let's say if they're sculling with both hands, what a lot of people do is they get to the front stops then they start putting their legs on and the blade is not in the water. So, the way I get them to think about it is to think of putting the blade in as they're still coming forward. Now you physically can't do that but that's the concept I talk about. So not getting there, then trying to get the blade in, because it's too late. So, squaring your blade early… With a square blade, right? So, I get them to square from about half slide forward, so the blade is fully squared. You are not getting there, squaring up, and put the blade in. Then you will miss water. […] In sweeping it's with the outside hand. Most people try to put the blade in with the inside hand. But you need to maintain contact with the outside hand. So, the main, key point for me is putting the blade in the water as it's still coming forward, that's what they need to think about. And then applying the legs. So, it's placement first, then leg drive.

This aspect was named *Catch and Leg Drive*. Although in the initial introduction, it sounded like the leg drive, including the power application, is more important—which would have put this aspect in the force-application category—in the coach's explanation, it becomes evident that the fine motor skills (e.g., hand movements) are indeed more important for the catch. Therefore, the aspect *Catch and Leg Drive* was assigned to the category *perceived movement precision*. It is noteworthy that this coach even aims to get athletes to initiate the catch during the recovery. The coach correctly says that physically the rowers cannot place the blades during the recovery; they also cannot wait to use their legs until the blade is placed as rowers must apply the legs before the blades touch the water (25, 35). Nevertheless, this coach attempts to promote an overcorrection to account for the common mistake of a late blade entry by telling his athletes to approach the water during the recovery.

Some aspects seem partially influenced by an organisation's leading ideas on rowing technique. For example, one coach mentioned that his coaching and assessing of *Phase Structure* depends on the organisation's guidelines wherever he currently coaches. Additional aspects in this category are related to gaining balance by setting the boat up with correct hand movements at the finish, correct back shape to prevent injuries, the length of the stroke, and precise movements at the catch to quickly find the connection between blades and the water.

### 3.2 Perceived force application

The second category is *perceived force application*. All aspects in this category relate to how, when, and where (i.e., with which muscle groups) to apply power. For this category, we look at a quote by Coach B: “[…] together with the velocity of the boat—that increases while overcoming inertia—to keep the grip on the blade, and the feet, until the boat pivots your hips and the boat, brings the hands [toward the body].”

Later in the interview, Coach B thoroughly described what she looks for when examining the execution of this aspect:

So, he must push his legs. I can see that when there is an oar bend. Then the question arises: How long does the connection hold? When there is no oar bend or only when the boat has already gone past the blade then he only got the connection at this point. I see it when the oar is straight. I also see it when there is white water around the blade. This is a sign that it is not the boat going past the blade but just the blade moving in the water. White water around the blade is like poison. […] And after we push our legs and then there is the linkage [of the upper body]. And I see it very easily when the legs are not moving, and the upper body is opening up. Then the performance is bad too. But he might actually have the connection, however, it is not on his feet, it is with the upper body, so too early, and therefore a waste of effort. Something that later in the drive would be more effective to push the boat further is used up already. […] The idea is to use the leg drive, the extension of the hips, and the arm draw as late as possible and as early as necessary. And when, at the catch, he immediately opens his hips, he only has the length of the leg drive. Or when he draws the arms at the catch he cannot hang at the finish until the velocity of the boat bends his elbows.

This aspect was named *blade during the drive* and assigned to the category *force application*. This is a more gross motor skill-based criterion, and the application of power is the main focus. In this category, most criteria are related to being connected to the water during the drive. The goal of the force application is to propel the boat with the movement instead of being inefficient and wasting energy with a blade that is not well connected to the water. In other aspects of this category, coaches discussed the importance of body tension and the correlation between body and boat movement.

### 3.3 Perceived rhythm and timing

The third category is *perceived rhythm and timing*. Generally, with this category, a coach might not be able to tell if the aspects are executed well when looking at a picture of individual athletes because the positions might be correct. However, considering the time frame of movements, mistakes can be spotted—usually spotted only in real-time. For the last category, we look at an example offered by Coach C:

So, the first thing I focus on in a crew boat is if their rhythm is the same. So, if they move together in the boat. And I would make them change that if it was not correct because that is what bothers my eyes the most. Someone who moves against the rhythm or not entirely with the rhythm.

His description of this aspect offers his perspective on the perfect and flawed execution of the discussed aspect:

So, a good performance is when the blades go in and out of the water simultaneously. And it is pretty easy to see when a blade goes in too early or too late. Or if a blade is peaking out while the other one is still in the water. So, check if this is executed well. Synchronicity is mostly blade work, I examine it by checking the blade work. […] It is the first thing that immediately jumps into the eye, and you just think: no, this cannot be. When this is not executed well you cannot say: all right, “same length” and “together with the legs”… It is the most important thing. When someone has a different timing, it is over.

The importance of timing in this aspect is evident. While all rowers might have the same movement, which could be the perfect stroke, their timing is off. Aspects that match this description have been assigned to the category *perceived rhythm and timing*. The most important aspects are the timing itself, especially of the blades at the catch and the finish, stroke rhythm, and the whole crew's synchronicity.

## 4 Discussion

The aim of this study was to explore how expert coaches assess athletes’ rowing technique during on-water technique sessions. Guided by the repertory grid technique, four semi-structured interviews were conducted with expert-level rowing coaches and content-analysed to determine their subjective technique criteria. The collected data provided insights into the coaches’ subjective criteria for technique evaluation, which can help novice and intermediate coaches to develop their coaches’ eye.

Interestingly, the aspects mentioned by the coaches as their initial foci for technique evaluation varied. This finding confirms the previously suggested differences between coaching styles and coaches’ criteria for the rowing technique (36, 37). The categories inductively established for the summarising Tables 1, 2 were *perceived force application*, *perceived movement precision*, and *perceived rhythm and timing*. The allocation of the mentioned aspects was sometimes not entirely conclusive for a few aspects, meaning that it was possible to assign them to multiple categories. However, it was found that force application, movement precision, and rhythm and timing aspects are all relevant to the coaches’ evaluation of athletes’ technique because all these categories were mentioned repeatedly in nearly every interview.

The mentioned aspects can be viewed in correspondence with biomechanical principles [for an overview, see (35)], as they are relevant to creating more propulsion, reducing resistance, or both. When juxtaposing our results to those of Legge et al. (38), it is noticeable that the coaches in our study consider aspects from all three themes that were also identified in their study—(1) getting the basics right, (2) targeting types of talent, and (3) complexity of performance. This makes sense when considering that expert coaches work with expert-level athletes instead of non-experts; therefore, assuming a high level of technical proficiency is warranted. Interestingly, of the mentioned aspects in the present study, only force-application aspects can be found in the most complex theme (i.e., complexity of performance). This suggests that this might be the most complex category of criteria requiring the most technical proficiency, and these aspects might be challenging to teach to technically or physiologically inferior athletes.

Decisions by expert coaches are highly nuanced and based on various factors such as personal experiences and (in)formal education (17). Hence, coaches’ perceptions of athletes are always influenced not only by their knowledge and perception of perfect technique but also by their previously acquired knowledge about athletes (39). According to the coaching model by Côté et al. (39), the coach's mental model of the athlete's potential determines the coach's approach to an athlete's training and what needs to be done to reach their goals. The discussed setting of a situation where the coach has no pre-existing knowledge about the observed athletes is a rare occurrence in most coaches’ day-to-day settings. Therefore, a coach's approach to an athlete of their team might differ from their approach to an athlete they see for the first time.

Many junior coaches orient themselves based on the technical models of sports organisations or world-class crews. Nevertheless, individual characteristics should be considered when assessing rowers, especially in teams with high heterogeneity of athletes’ physical characteristics. During the interview, one of the coaches pointed out that when working with older recreational rowers, who may have less strength and flexibility, it is necessary to adjust expectations compared to athletes in their prime. This demonstrates the need to adapt the technique to an athlete's potential, a factor rarely considered when coaches are questioned about the ideal technique for highly skilled rowers. Physical characteristics such as body height, muscle mass, or endurance capacity must be considered when a coach creates expectations for an individual athlete, similar to athlete age considerations. Based on these characteristics, a junior coach might have to assess differently than a senior-level coach. Moreover, as an outdoor sport, rowing is influenced by several varying external factors, such as water current, wind, waves, (water) temperature, and motorboat wake (40). Environmental factors, therefore, must also be considered when coaching and assessing rowers.

Differences in technique in various boat, age, and weight categories were also not thoroughly discussed in the present study. Some coaches might have been generalising criteria that are mainly relevant to either sculling or sweeping for both techniques because their coaching is mainly in sculling or sweeping. However, in several smaller rowing federations (like the Swiss Rowing Federation), most coaches are exposed to both because, unlike in other bigger rowing federations, the sculling and sweeping teams are not separated and coached by the same coaches.

### 4.1 Limitations and future research

It is noteworthy that the population of the participants of this pilot study is relatively small due to the high specialisation in the field of expert coaches in rowing. The similarity of the rowing technique criteria might be influenced by the fact that the coaches interviewed came from the same rowing federation. This, in turn, would make the data especially suitable for rowers and coaches from the same context (i.e., the same federation). It would be interesting to compare coaches from different nations and backgrounds to learn about prevalent views in teams worldwide, instead of only discovering individual views/idiosyncrasies [see also (38)]. A large and diverse sample could lead to more conclusive findings about technique assessment with high generalisability. For example, to determine which aspects are most relevant for expert coaches. One option could be moving from a qualitative approach to a more quantitative approach by implying standardised questionnaires. However, this can only be done correctly after enough data are collected. Nevertheless, the initial foci will hardly ever lead to international consensus because even the coaches’ criteria within our relatively homogenous expert sample were already subjective and noticeably varied. What can be determined from such a study is the tendency with which aspects matter to the majority of coaches. We hypothesise that we would find similar aspects with a slightly higher heterogeneity and different terms in different coaching settings (e.g., other national teams). With the current knowledge, there seems to be a certain degree of freedom for a couple of aspects where personal style preferences come into play. Crews can use technique models as a framework for their rowing technique, but even elite rowers may individually adapt their execution of the rowing stroke (25). This can be equally or even more efficient than a “perfect” execution of the model technique.

Additionally, the categories in Tables 1, 2 overlap, and some criteria may belong to more than one category. This is owed to the fact that the rowing stroke must be executed well in its entirety for ideal performance, and distinct aspects influence each other, which creates a causal relationship between aspects. The coding was done according to the coaches’ emphasis when discussing the mentioned criteria. However, the data analysis might be skewed due to subjective interpretation of the researchers. For example, the researchers’ preconceptions or prior knowledge might influence the interpretation and categorisation of the data.

Moreover, countless factors, such as anthropometry, boat material, and environmental factors, among others, influence the ideal performance of the rowing stroke (25). Accordingly, the “perfect technique” description cannot be entirely conclusive and may be adjusted slightly by different experts. As the interviews showed, the different styles in international rowing may also depend on the coaches and their concept of rowing technique.

Technical aids to measure aspects of the rowing technique, such as a “SpeedCoach” (Nielsen Kellerman, Boothwyn, PA, USA) and real-time force and angle measurement tools (e.g., Nielsen Kellerman EmPower Oarlock or the FlexOmega system) are becoming more straightforward and more affordable (41). It can be assumed that these innovations will increasingly assist and supplement the coaches’ perspective. Through physical measurement, they can directly measure aspects that coaches without these tools can only assume from the displayed technique execution—such as force curves and stroke length.

Following the repertory grid technique (31), it would also be interesting if the coaches rated athletes according to their own criteria. Getting several coaches to judge the same athletes based on their criteria and comparing their scores for the same athletes would address the objectivity, validity, and reliability of the subjective criteria mentioned by the coaches [for an example of the application of the repertory grid technique, see (30)].

### 4.2 Conclusion

The present study aimed to explore expert-level coaches’ subjective criteria when assessing the technique of on-water rowing. Four coaches with international coaching experience were interviewed to obtain the required data. The coaches’ subjective technique criteria gave an insight into the broadness of coaching styles in rowing. Evidence from the present study shows that *perceived movement precision*, *perceived force application*, and *perceived rhythm and timing* are important in the assessment of technique by rowing coaches. These findings can be particularly helpful for novice coaches in improving their ability to teach rowing techniques by building on the established criteria of expert coaches.

## Data Availability

The raw data supporting the conclusions of this article will be made available by the authors, without undue reservation.
