# Supplementary material for: Insights from expert coaches on technical performance evaluation in rowing: a pilot study
Source: Front Sports Act Living. 2024 Oct 10;6:1448797. doi: 10.3389/fspor.2024.1448797 (PMC11499098; doi:10.3389/fspor.2024.1448797)
Supplement: Supplementary file 1 [file Table1.docx]

Supplementary Material

Insights from expert coaches on technical performance evaluation in rowing: a pilot study

Erik Baumann and Michael J. Schmid*

***Correspondence:** Michael J. Schmid: michael.j.schmid@unibe.ch

# Interview Guide for Expert Interviews

| **Interview part** | **Description** |
| --- | --- |
| Introduction | “Thank you for participating in this project. My supervisors and I appreciate your effort concerning this study and hope you will enjoy the interview.” |
| Information | “Before we can start with the interview, we need to make sure that you are well-informed about the purpose of the study, the approach of the interview and how we deal with the data.  The aim of the project is, finally, to develop an understanding of elite-level rowing coaches’ approach to technique assessment.  The purpose of the interview is to find out what coaches with less experience should look at during technique training or assessment of rowers with the purpose of selection. That means we are looking for the various aspects that should be included and combined to provide a rower’s technique profile. Moreover, we want to be able to judge these aspects on their quality. This means we need to know when an aspect of the technique is performed well and when not.  Just to make sure: we are ignoring physiology, and we are assuming rigging is perfectly adjusted.” |
| Informed consent | “So, at this point, I will ask you to read the informed consent letter and sign it if you accept its terms and conditions.” |
| Start recording | “From this point in time we start recording the interview. Is that okay? Moreover, I might write down short notes to better structure the interview.” |
| Years of competing | “Before we start with the questions regarding rowing technique, we will start by talking about your personal experience as an athlete and coach in rowing. We will start by talking about your personal experience as a rowing athlete. How many years did you compete in the sport? And what would you say was your biggest success as an athlete?” |
| Years of coaching | “And then coaching. How did you get into this aspect of the sport, and how long was/is your coaching career?” |
| Levels of coaching | “How many years did you coach at the club level? How many internationally? What is the biggest success crews coached by you have achieved?” |
| Aspects of technique | “Imagine yourself watching a newly formed crew of highly skilled rowers [in the context of a club or national team] or a single sculler that you have not seen rowing before. Your goal for this session is to evaluate their technical skill level. What are some things you would consider?”  (e.g., body positions – bladework – length – angles – boat run – catch – finish – drive – recovery – fluidity – rhythm – back shape – synchronisation) |
| Quality of the aspects | “If you consider …; what is the perfect performance? And what would be the worst or flawed performance? Explain, clarify, and describe an ideal execution of this aspect.” |
| Additions | “We talked about a lot of points. Is there anything that you would like to add? Did we forget about an aspect?”  “Anything specific for sweeping/sculling?” |
| End | “We are now at the end of the interview. When ready, we will send you an overall summary of the data we collected from all the coaches to check whether everything we discussed is adequately described. At that point, additions are still welcome.” |
